# A Potential Nutraceutical Candidate Lactucin Inhibits Adipogenesis through Downregulation of JAK2/STAT3 Signaling Pathway-Mediated Mitotic Clonal Expansion

**DOI:** 10.3390/cells9020331

**Published:** 2020-01-31

**Authors:** Xin Wang, Min Liu, Guo He Cai, Yan Chen, Xiao Chen Shi, Cong Cong Zhang, Bo Xia, Bao Cai Xie, Huan Liu, Rui Xin Zhang, Jun Feng Lu, Meng Qing Zhu, Shi Zhen Yang, Xin Yi Chu, Dan Yang Zhang, Yong Liang Wang, Jiang Wei Wu

**Affiliations:** Key Laboratory of Animal Genetics, Breeding and Reproduction of Shaanxi Province, College of Animal Science and Technology, Northwest A&F University, Yangling 712100, China; 18829353049wx@nwafu.edu.cn (X.W.); mliu0903@nwafu.edu.cn (M.L.); caiguohe@nwsuaf.edu.cn (G.H.C.); chenyan0806@nwsuaf.edu.cn (Y.C.); xcshi1105@nwsuaf.edu.cn (X.C.S.); zhangcongcong@nwafu.edu.cn (C.C.Z.); imed23@nwafu.edu.cn (B.X.); xiebaocai2017@nwafu.edu.cn (B.C.X.); huanliu150808@nwafu.edu.cn (H.L.); ruiznxinzhang@nwafu.edu.cn (R.X.Z.); lujunfeng@nwafu.edu.cn (J.F.L.); zhumengqing@nwafu.edu.cn (M.Q.Z.); yangshizhen@nwsuaf.edu.cn (S.Z.Y.); chuxy0919@nwafu.edu.cn (X.Y.C.); zhangdanyang@nwafu.edu.cn (D.Y.Z.); yongliang.wang@nwafu.edu.cn (Y.L.W.)

**Keywords:** lactucin, adipogenesis, JAK2/STAT3, mitotic clonal expansion (MCE), adipocyte, obesity

## Abstract

The prevalence of obesity has increased dramatically worldwide in the past ~50 years. Searching for safe and effective anti-obesity strategies are urgently needed. Lactucin, a plant-derived natural small molecule, is known for anti-malaria and anti-hyperalgesia. The study is to investigate whether lactucin plays a key role in adipogenesis. To this end, in vivo male C57BL/6 mice fed a high-fat diet (HFD) were treated with 20 mg/kg/day of lactucin or vehicle by gavage for seven weeks. Compared with vehicle-treated controls, Lactucin-treated mice showed lower body mass and mass of adipose tissue. Consistently, in vitro 3T3-L1 cells were treated with 20 μM of lactucin. Compared to controls, lactucin-treated cells showed significantly less lipid accumulation during adipocyte differentiation and lower levels of lipid synthesis markers. Mechanistically, we showed the anti-adipogenic property of lactucin was largely limited to the early stage of adipogenesis. Lactucin-treated cells fail to undergo mitotic clonal expansion (MCE). Further studies demonstrate that lactucin-induced MCE arrests might result from reduced phosphorylation of JAK2 and STAT3. We then asked whether activation of JAK2/STAT3 would restore the inhibitory effect of lactucin on adipogenesis with pharmacological STAT3 activator colivelin. Our results revealed similar levels of lipid accumulation between lactucin-treated cells and controls in the presence of colivelin, indicating that inactivation of STAT3 is the limiting factor for the anti-adipogenesis of lactucin in these cells. Together, our results provide the indication that lactucin exerts an anti-adipogenesis effect, which may open new therapeutic options for obesity.

## 1. Introduction

Obesity is a global health issue. According to the World Health Organization (WHO), 39% of women and 39% of men aged 18 and over were overweight [1,2]. It is a risk factor for the development of different conditions such as type 2 diabetes mellitus, coronary thrombosis, cancer, and osteoarthritis [3]. Currently, five FDA-approved medications for obesity treatment could be classified into two types, pancreatic lipase inhibitors to reduce intestinal fat absorption, and anorectics to suppress appetite. However, most of them have undesirable adverse effects [4]. Thus, there is still a desperate demand for effective and safe candidates to get obesity under control. Recently, researchers have discovered that natural small molecules such as triterpenoid (celastrol) [5], natural phenol (resveratrol) [6], alkaloid (capsaicin) [7,8] and sesquiterpene lactones (Artemisinin) [9] have the anti-obesity properties. Sesquiterpene lactones are a large and structurally diverse group of plant second metabolites [10] with distinctive biological activities, including gastric cytoprotective effects [11], anti-migraine [12], antiviral and antimicrobial activities [13,14], anti-tumor [15] and neurotoxic effect [16].

Cellular studies of obesity involve hyperplasia (cell number increase) and hypertrophy (cell size increase). Hypertrophy requires differentiation of preadipocytes to mature adipocytes, which is induced by MDI and accompanied by the regulation of various transcription factors, including C/EBP α, C/EBP β and PPAR γ [17]. The expression of these transcription factors marked terminal adipocyte differentiation and triglyceride-synthesis [18]. In vivo, these transcription factors also play vital roles in lipid deposition [19]. Several studies showed that sesquiterpene lactone affects the expression of C/EBP β, C/EBP α, and PPAR γ, and therefore regulating the adipogenesis of adipocytes [20,21,22]. C/EBP β is the first transcription factor induced after exposure of the preadipocytes to the differentiation cocktail and its activity is thought to mediate the expression of PPAR γ and C/EBP α [23]. In addition, C/EBP β has been shown to be a direct target site of STAT3 in preadipocytes, and the JAK2/STAT3 signaling pathway plays an important role in lipid synthesis. Adipocytes produce hormones that act in an endocrine manner and engage the JAK/STAT signaling pathway in its target tissues [24]. The role of STAT3 in preadipocytes has been studied, showing that STAT3 affects the differentiation of 3T3-L1 preadipocyte. Also, p-STAT3 has been shown to promote preadipocyte differentiation by modulating the progress of mitotic clonal expansion [25].

Lactucin, a sesquiterpene lactone, is one of the most abundant active constituents in endive. It has multiple biological activities including anti-malaria and anti-hyperalgesia [26]. Previous studies have shown that lactucin regulates energy metabolism by modulating the mRNA expression of cytochrome P450 in hepatocytes [27]. However, the effect of lactucin on obesity has not yet been studied. Based on these indications, we predict that lactucin may possess the ability to inhibit adipogenesis through regulation of the expression of C/EBP β, C/EBP α and PPAR γ.

Altogether, in the present study, we showed that lactucin prevents HFD-induced obesity in mice. We then validated this inhibitory effect in the 3T3-L1 cell in vitro.

## 2. Materials and Methods

### 2.1. Ethics Statement

The animal protocol was reviewed and approved by the Animal Care and Use Committee of Northwest A&F University (ID: 20190412002) and performed in accordance with animal welfare and ethics.

### 2.2. Animals and Diets

Six-week-old male C57BL/6 mice were purchased from the animal center of Xi’An Jiao Tong University (Xi’an, China). Mice were acclimated for two weeks in the animal facility of Northwest A&F University under a high-fat diet (composed of 26.2% protein, 34.9% fat, D12492, Xie Tong Corp., Nanjing, China) feeding. At the age of seven weeks, mice were received 20 mg/kg/day of lactucin [28] (SHANGHAI ZZBIO, Shanghai, China) or vehicle by gavage, with free access to food and water in a 12-h light/dark animal facility.

### 2.3. Glucose and Insulin Tolerance Test

For the GTT, C57BL/6 mice were overnight fasted (15 h) after five weeks of treatment and injected with 2 g/kg body weight D-glucose into the peritoneal cavity. Blood samples were obtained from the tail vein of each mouse and levels of glucose were determined at indicated time points (0, 15, 30, 60, 90, and 120 min). For the ITT, 5 h fasted mice (from 6 am to 11 am) after six weeks of treatment, the glucose concentrations were measured at 0 and 15, 30, 60, 90, and 120 min after injection of human insulin (Eli Lilly) at 1 U/kg body weight. Plasma glucose concentration was measured using a YuYue Ultra Glucose Meter (LifeScan, Inc., Milpitas, CA, USA).

### 2.4. Histological Processing and Morphological Evaluation

Tissue fragments were fixed in buffered formalin, then paraffin-embedded for hematoxylin-eosin staining. Image J was used for adipocyte diameter measurements. The distribution of cell size was expressed as the percentage of total counted adipocytes. An average value across five non-overlapping images (five/section) was calculated for each group (*N* = 5). Staining and imaging procedures were done randomly.

### 2.5. Assessment of Cell Viability

3T3-L1 cells were plated in 96-well plates, and each well was seeded with about 10,000 cells. Cells were incubated in 100 μL of DMEM supplemented with 10% FBS, and the time point was designated as 0 h. After 4 h of incubation at 37 °C, cells were attached to the plate. CCK8, a tetrazolium reagent [2-(4-indophenyl)-3-(4-nitrophenyl)-5-(2,4-disulfophenyl)-2H-tetrazolium monosodium salt] were added at the time point of 24 h. DMEM/F12 containing 10% CCK8 medium was substituted for previously cultured medium, and every well had 100 μL fresh medium. The cells were incubated with CCK8 reagent for 2 h at 37 °C. The staining intensity was measured in terms of absorbance at 450 nm.

### 2.6. Cell Culture and Adipocyte Differentiation Induction

3T3-L1 preadipocytes obtained from American Type Culture Collection (ATCC) were maintained in DMEM (SH30022.01, HyClone, CT, USA) containing 10% fetal bovine serum (Z7186FBS-500, ZETA LIFE, Menlo Park, CA, USA). To induce differentiation, 2-day post confluent 3T3-L1 cells (designated at day 0) were cultured in DMEM containing 10% FBS, 0.5 mM 3-isobutyl-1-methylxanthine (IBMX) (I7018, Sigma-Aldrich, St. Louis, MO, USA), 1 μM dexamethasone (D4902, Sigma-Aldrich, MO, USA), and 10 μg/mL insulin (91077C, Sigma-Aldrich, St. Louis, MO, USA) for two days. Cells were then maintained in DMEM containing 10% FBS and 10 μg/mL insulin for the rest of the culture.

### 2.7. Oil Red O Staining and Lipid Quantification

Oil Red O staining was performed on day 8 for visualization of the accumulated lipid droplets in the differentiated adipocytes. After induction of adipocyte differentiation, cells were washed three times with PBS, fixed with 4% formaldehyde for 30 min at room temperature, and then rinsed with phosphate buffered saline. 3T3-L1 cells were then stained with Oil Red O solution for 30 min at room temperature. After removing the staining solution, the stained cells were washed at least three times with phosphate-buffered saline. The stained lipid droplets were visualized by light microscopy. The stained lipid was dissolved in isopropanol and quantified with a Microplate Reader at λ = 510 nm.

### 2.8. Quantitative Real Time-PCR

Total RNA was extracted from the cells and tissues using Trizol reagent, RNA was subjected to reverse transcription using the cDNA synthesis kit Super Script II. Quantitative real time-PCR analysis for adipogenic genes was performed on a real time-PCR system. Gene expression was detected using SYBR Green, and the relative gene expression was determined by normalizing to the reference gene, β-actin, with the relative quantitative method. The sequences of the primers corresponding to mouse adipogenic genes that were analyzed in this study are presented as Table S1.

### 2.9. Western Blot Analysis

Tissues were homogenized with glass Tenbroeck tissue grinders (Kimble Chase) on ice and lysed with lysis buffer (Beyotime Technologies, Beijing, China) containing protease and phosphatase inhibitor cocktails at 4 °C for 1 h and followed by centrifugation at 13,000× *g* for 10 min at 4 °C. Cultured cells were lysed in lysis buffer containing protease and phosphatase inhibitor cocktails on ice. Crude lysates were centrifuged at 13,000× *g* for 10 min at 4 °C. Total protein concentration from the resultant supernatant was determined by a BCA protein assay kit (Biobox, Biotech, Nanjing, China). 20 μg protein samples were separated by sodium dodecyl sulfate-polyacrylamide gel electrophoresis and transferred to a polyvinylidene difluoride membrane (Beyotime Technologies, Beijing, China) by electroblotting. Membranes were incubated overnight at 4 °C with primary antibodies. After washing, the secondary antibody was added and incubated 2 h at room temperature and protein bands were visualized by ECL Plus (310212, ZETA LIFE, Menlo Park, CA, USA). Densitometric quantitation was performed using a Sagecreation imaging system with Sagecreation Quantity One software (Sagecreation, Beijing, China). Antibodies used for immunoblotting were listed in Table S2.

### 2.10. Cell Cycle Analysis

3T3-L1 cells were treated with differentiation medium in the presence or absence of lactucin for 24 h. Next, 10 000 cells from each experimental condition were fixed overnight with 70% ethanol at 4 °C, and then incubated with 10 μg mL^−1^ of RNase A and 50 μg mL^−1^ of propidium iodide for 30 min at room temperature in the dark. DNA content was measured with a Guava easyCyte™ Flow Cytometer (Merck Millipore, Billerica, MA, USA), and analysis of the cell cycle was performed using the FCS Express 4 Flow Cytometry software (De Novo Software, Los Angeles, CA, USA).

### 2.11. Statistical Analysis

Data were presented as mean ± standard error (SEM). Inter-group differences between two groups were assessed by an unpaired Student’s *t*-test using GraphPad Prism 6 software (GraphPad Software, Inc., La Jolla, CA, USA). While one-way analysis of variance (ANOVA) was used for comparison amongst multiple groups using PASW Statistics 20 (SPSS, Chicago, IL, USA). A *p* value < 0.05 was considered statistically significant.

## 3. Results

### 3.1. Lactucin Treatment Prevents HFD-Induced Obesity and Hyperglycemia in Mice

**Figure 1:**
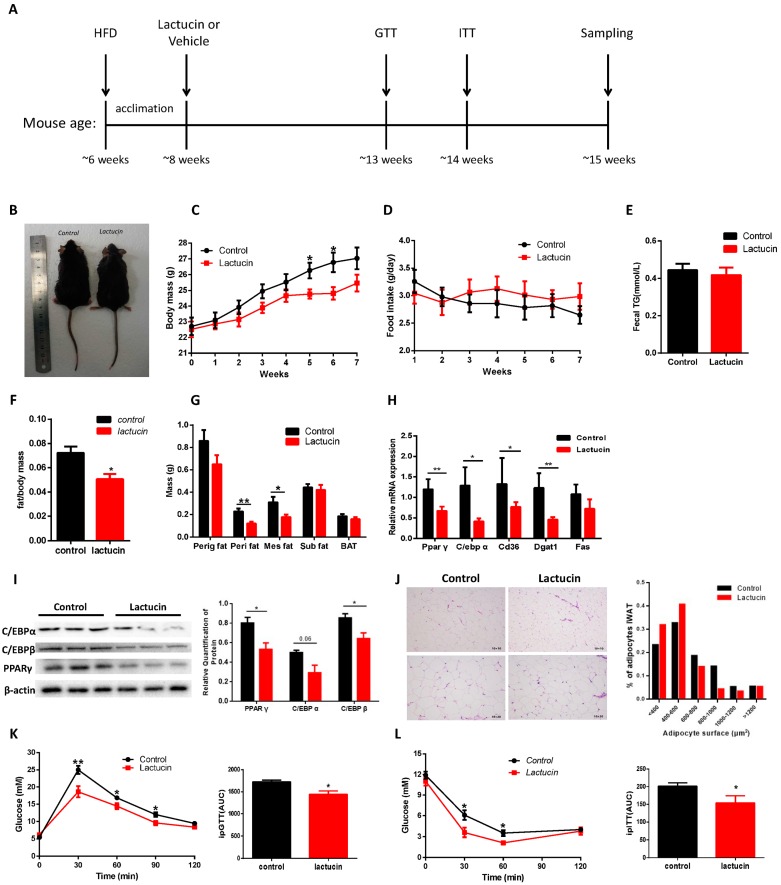
Lactucin treatment prevents HFD-induced obesity in mice. Male C57BL/6 mice fed a high-fat diet (HFD) were treated with 20 mg/kg/day of lactucin or vehicle by gavage starting from seven weeks of age. (**A**) Schematic diagram of mice treatment. (**B**) Macroscopic views of control and treated mice. (**C**) Weekly body mass (*n* = 7). (**D**) Weekly food intake (*n* = 7). (**E**) Fecal triglyceride (TG) levels (*n* = 7). (**F**) Adipose tissue weight to body weight ratio (*n* = 7). (**G**) Mass of fat depots (perigonadal fat, perirenal fat, mesenteric fat, subcutaneous fat, and BAT) (*n* = 7). (**H**) The mRNA levels of Ppar γ, C/ebp α, Cd36, Dgat1 and Fas in adipose tissue (*n* = 5). (**I**) Protein levels of PPAR γ, C/EBP α, and C/EBP β, as shown by Western blot (*n* = 3). (**J**) Histology of iWAT after seven weeks of treatment. (**K**) Blood glucose levels during glucose tolerance tests (*n* = 7). (**L**) Blood glucose levels during insulin tolerance tests (*n* = 7). Values are mean ± S.E.M. * *p* < 0.05, ** *p* < 0.01.

To detect the effect of lactucin on adipogenesis in vivo, male C57BL/6 mice fed a high-fat diet (HFD) were treated with 20 mg/kg/day of lactucin or vehicle by gavage starting from seven weeks of age. Compared with vehicle-treated controls, lactucin-treated mice showed lower body mass and lower weight gain throughout the observation period of seven weeks (*p* < 0.05) (Figure 1B,C). These bodyweight changes were neither caused by a change in food intake nor in fat absorption in the intestine since food intake and fecal TG content were indistinguishable in the vehicle and lactucin-treated groups (*p* < 0.05) (Figure 1D,E). After seven weeks of lactucin treatment, lower body weight in lactucin-treated mice resulted from the decreased fat mass and a lower fat/body mass ratio (*p* < 0.05) (Figure 1F,G). At the same time, adipose tissue was subjected to histological analysis, showing significantly smaller adipocytes, in lactucin-treated groups than that of controls (Figure 1J). The mRNA and protein levels of PPAR γ, C/EBP α, and C/EBP β in lactucin-treated adipose tissue were markedly lower than that of controls (*p* < 0.05) (Figure 1H,I). Next, we tested whether lactucin regulates HFD-induced glucose tolerance and insulin sensitivity. As shown in Figure 1K,L, lactucin prevents HFD-induced glucose intolerance as well as insulin resistance (*p* < 0.05). Also, plasma levels of TG and LDL were measured. The results showed significantly lower levels of TG and LDL in lactucin-treated mice than in controls (Figure S1E,F). Meanwhile, the effect of lactucin on hepatic lipogenesis were measured. Compared to controls, lactucin treatment significantly reduced liver size and weight (Figure S2A,B). We then examined the protein levels of Acetyl-CoA carboxylase (ACC) and Fatty acid synthase (FAS) in the liver, showing that lactucin-treated livers had lower levels of the protein expressions of both enzymes (Figure S2C).

Given the safety of the compound, treated C57BL/6 mice tolerated lactucin well during the seven weeks of compound administration with no apparent side effects. Plasma activities of the liver transaminases ALT and AST were lower in lactucin-treated mice than that of controls (Figure S1A,B), suggestive of normal or even improved liver function. Similarly, blood urea nitrogen (BUN) and creatinine (CREA) concentrations were comparable between both groups of mice (Figure S1C,D), providing evidence for normal kidney function. Together, these results show that lactucin treatment protects mice against obesity and hyperglycemia without obvious side effects.

### 3.2. Lactucin Treatment Inhibits Adipogenesis of 3T3-L1 Cells

**Figure 2:**
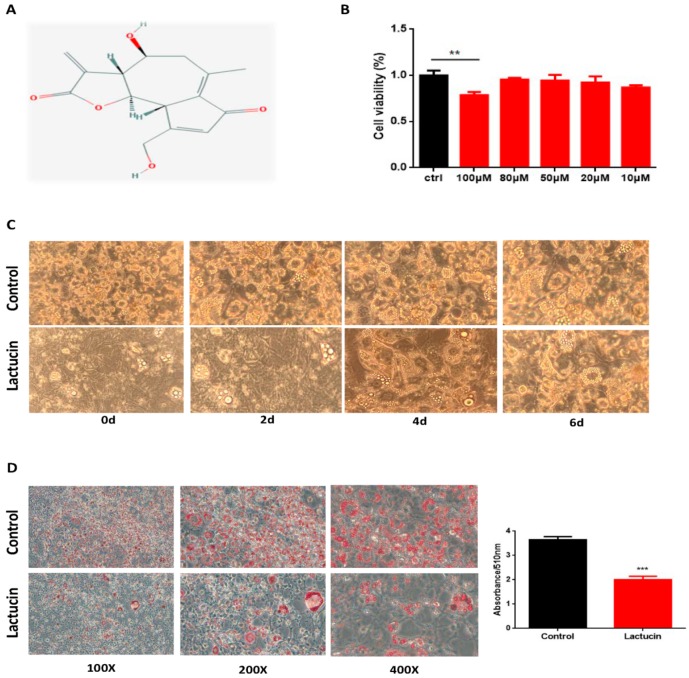
Inhibition of lactucin on lipid accumulation in 3T3-L1 adipocytes. (**A**) The molecular formula of lactucin. (**B**) 3T3-L1 preadipocytes were treated with the indicated concentrations of lactucin for 48 h. Cell proliferation was then determined by the CCK-8 assay (*n* = 5). 3T3-L1 preadipocytes were induced to differentiate with the standard regimen (IBMX, dexamethasone, and insulin; MDI) and 20 μM lactucin was added 0–6, 2–6, 4–6 and 6–8 days of adipogenesis. (**C**) Morphological observation and (**D**) Oil Red O staining of 3T3-L1 adipocytes photographed using a microscope (×100). Lipid droplets were stained in red. And absorbance of extracted ORO accumulated in lipid droplets of 3T3-L1 adipocytes was measured spectrophotometrically at 510 nm. Values are mean ± S.E.M. ** *p* < 0.01, *** *p* < 0.001.

To further investigate the effect of lactucin on adipogenesis, 3T3-L1 cells were treated with different concentrations of Lactucin (100 μM, 80 μM, 50 μM, 20 μM, and 10 μM). Figure 2A is the molecular formula of lactucin. First, a CCK-8 assay was performed to examine whether lactucin has an influence on cell viability. The results revealed lactucin had no obvious cytotoxicity to cells at a concentration of ≤ 80 μM (*p* < 0.05) (Figure 2B). Then, 20 μM of lactucin was used for the following experiments. To examine the effect of lactucin on different stages of adipogenesis of 3T3-L1 cells, post-confluent 3T3-L1 preadipocytes were treated with 20 μM of lactucin for 0–6, 2–6, 4–6, 6–8 days. Adipogenesis was visualized with Oil Red O staining, showing significantly less lipid accumulation in early lactucin-treated cells than that of controls (*p* < 0.05) (Figure 2C,D), late-stage treatment from day 6 had no effect on adipogenesis, suggesting that early, but not late-stage, lactucin treatment efficiently suppresses adipogenesis of 3T3-L1 cells.

### 3.3. Lactucin Treatment Inhibits Adipogenesis of 3T3-L1 Cells by Downregulation of TG Synthesis

**Figure 3:**
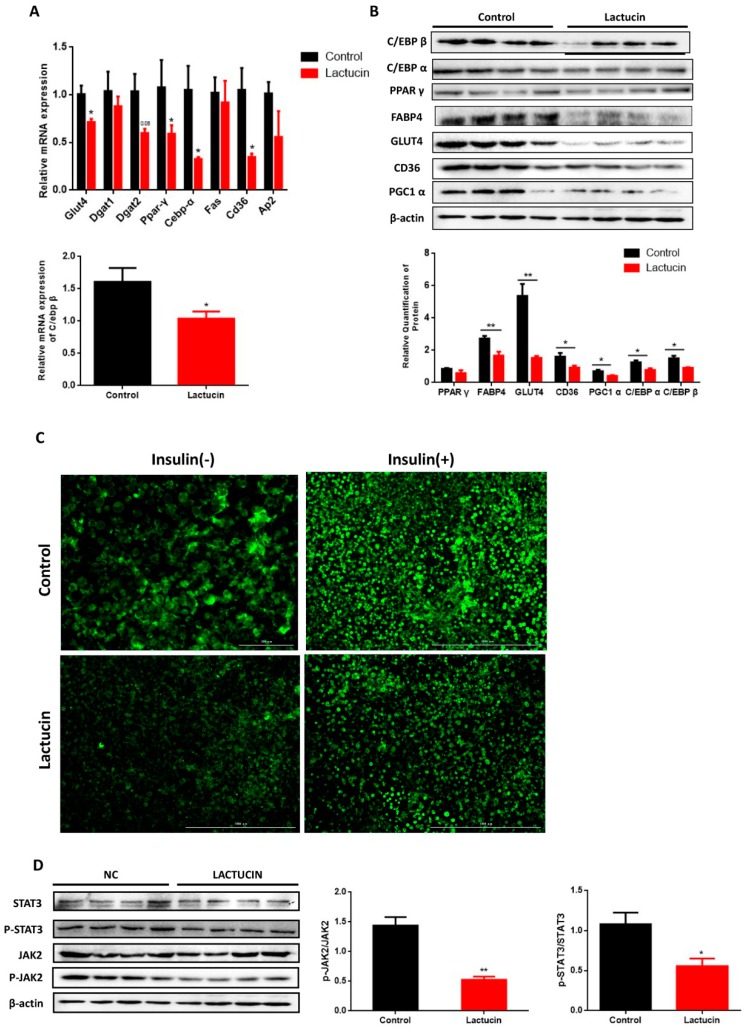
The effect of lactucin on the expression of adipogenic transcription factors and adipogenic genes in 3T3-L1 adipocytes. 3T3-L1 preadipocytes were cultured in the differentiation medium containing 20 μM lactucin. (**A**) The mRNA levels of lipid metabolism genes in 3T3-L1 adipocytes (*n* = 6). (**B**) The protein levels of lipid metabolism genes in 3T3-L1 adipocytes (*n* = 4). (**C**) Glucose uptake as shown by 2-NBDG. (**D**) The effect of lactucin on the JAK2/STAT3 signaling pathway (*n* = 4). The immunoblot analysis of the JAK2/STAT3 signaling pathway in 3T3-L1 adipocytes (*n* = 4). Values are mean ± S.E.M. * *p* < 0.05, ** *p* < 0.01.

Next, we try to detect whether less lipid accumulation in lactucin-treated cells was attributed to lower levels of TG synthesis. Levels of key transcription factors PPAR γ, C/EBP β, and C/EBP α, as well as key regulators including Dgat2, Cd36, Glut4, and Fabp4 of TG synthesis were measured. Lactucin-treated cells showed lower levels of PPAR γ and C/EBP α than that of controls both in the transcriptional level and in the translational level (*p* < 0.05) (Figure 3A,B). Consistent with this, significantly reduced mRNA and protein levels of Dgat2, Fabp4, Cd36 and Glut4 were also observed in lactucin-treated cells than in controls (*p* < 0.05) (Figure 3A,B). Glucose is the main substrate for TG synthesis in cultured cells [29]. Glucose uptake was directly measured by 2-NBDG (a fluorescent tracer used for monitoring glucose uptake into living cells). Lactucin-treated cells showed markedly lower glucose uptake than that of control cells (*p* < 0.05) (Figure 3C). Together, these results suggest that lactucin treatment suppresses TG synthesis.

### 3.4. Reduced TG Synthesis in Lactucin-Treated 3T3-L1 Cells Accompanies Downregulation of the JAK2/STAT3 Signaling

To further explore the underlying mechanism of lactucin-mediated adipogenesis, the upstream of PPAR γ and C/EBP α were traced. JAK2/STAT3 was widely accepted as one of the main pathways regulating PPAR and C/EBP family transcription factors [30]. We, therefore, tested whether lactucin affects the activation of JAK2 and STAT3 in 3T3-L1 cells. Our results showed significantly lower levels phospho-JAK2 and phosphor-STAT3 in lactucin-treated cells than in controls (*p* < 0.05) (Figure 3D), suggesting lactucin inhibits activation of JAK2/STAT3 signaling pathway, which may be one of the potential mechanisms of its anti-adipogenic effect. Consistently, we found a similar pattern of JAK2/STAT3 in the lactucin-treated adipose tissue when compared to controls in mice (Figure S2D).

### 3.5. Lactucin-Induced Downregulation of STAT3 Subsequently Promotes G0/G1 Phase Arrest, Therefore Inhibiting Mitotic Clonal Expansion

**Figure 4:**
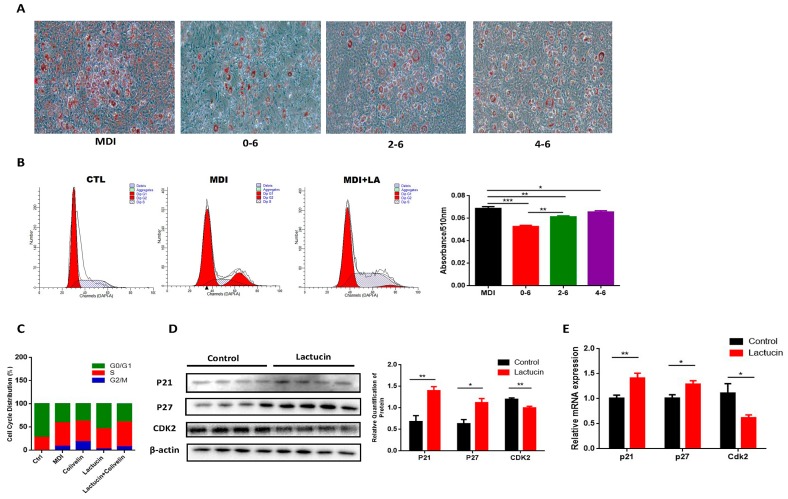
Lactucin promotes G0/G1 phase arrest through regulation of p21, p27, and CDK2. The 3T3-L1 preadipocytes were induced to differentiate with MDI and treated with 20 μM of lactucin for indicated times. (**A**) Morphological observation and Oil Red O staining of 3T3-L1 adipocytes photographed using a microscope (×100). Lipid droplets were stained in red. The absorbance of extracted ORO accumulated in lipid droplets of 3T3-L1 adipocytes was measured spectrophotometrically at 510 nm. (**B**) The cell cycle analysis using flow cytometry. (**C**) Percentage of cells in G0/G1, S and G2/M phases measured using FlowJo V10 software. (**D**,**E**) The mRNA (*n* = 6) and protein levels (*n* = 4) of genes related to cell cycle progression. Values are mean ± S.E.M. * *p* < 0.05, ** *p* < 0.01, *** *p* < 0.001.

Activation of STAT3 by phosphorylation is reported to promote the adipogenesis of 3T3-L1 cells via modulating the progress of mitotic clonal expansion (MCE), a proliferative phase that occurs in the early stage of adipogenesis [25,31,32]. Also, given the fact that we showed early lactucin treatment sufficiently suppresses adipogenesis (Figure 2B,C). We predicted that lactucin exerts its anti-adipogenic effect through the downregulation of STAT3-mediated mitotic clonal expansion. To test it, lactucin (20 μM) was added during days 0–6, 2–6, 4–6 of adipogenesis. Compared to late-stage treatment (days 4–6), early lactucin treatment significantly inhibited lipid accumulation in adipocytes (*p* < 0.05), with a maximum inhibition at 0–6 days (*p* < 0.05) (Figure 4A). The cell cycle changes were analyzed by flow cytometry, the result showed lactucin treatment effectively arrested the cell cycle at the G0/G1 phase (Figure 4B,C), preventing the mitotic clonal expansion of cells. We examined the effects of lactucin on the cell cycle in differentiated 3T3-L1 cells. Cyclin-dependent kinases (CDK) are a cell cycle-regulatory protein in G0/G1 phase, while p21 and p27 are inhibitors of CDK that modulate G1 phase arrest [33]. Consistently, expression levels of the cycle-regulatory protein in the G0/G1 phase cyclin-dependent kinases (CDK) 2 was significantly reduced, consistent with elevated levels of CDK inhibitors p21 and p27 (*p* < 0.05) (Figure 4D,E). Together, these results demonstrate that lactucin treatment upregulated the expression of p21 and p27, with a consequent downregulation of CDK2. Inhibition of CDK2 promotes G0/G1 arrest, therefore preventing mitotic clonal expansion in these cells. Together, these results showed that lactucin suppresses MDI-induced cell proliferation in 3T3-L1 cells during adipogenesis, suggesting that Lactucin induces G0/G1 phase arrest and inhibits CDK2 expression via enhancing p21 and p27 expression.

### 3.6. Pharmacological STAT3 Activator Reverses the Anti-Adipogenic Effect of Lactucin on 3T3-L1 Cells

**Figure 5:**
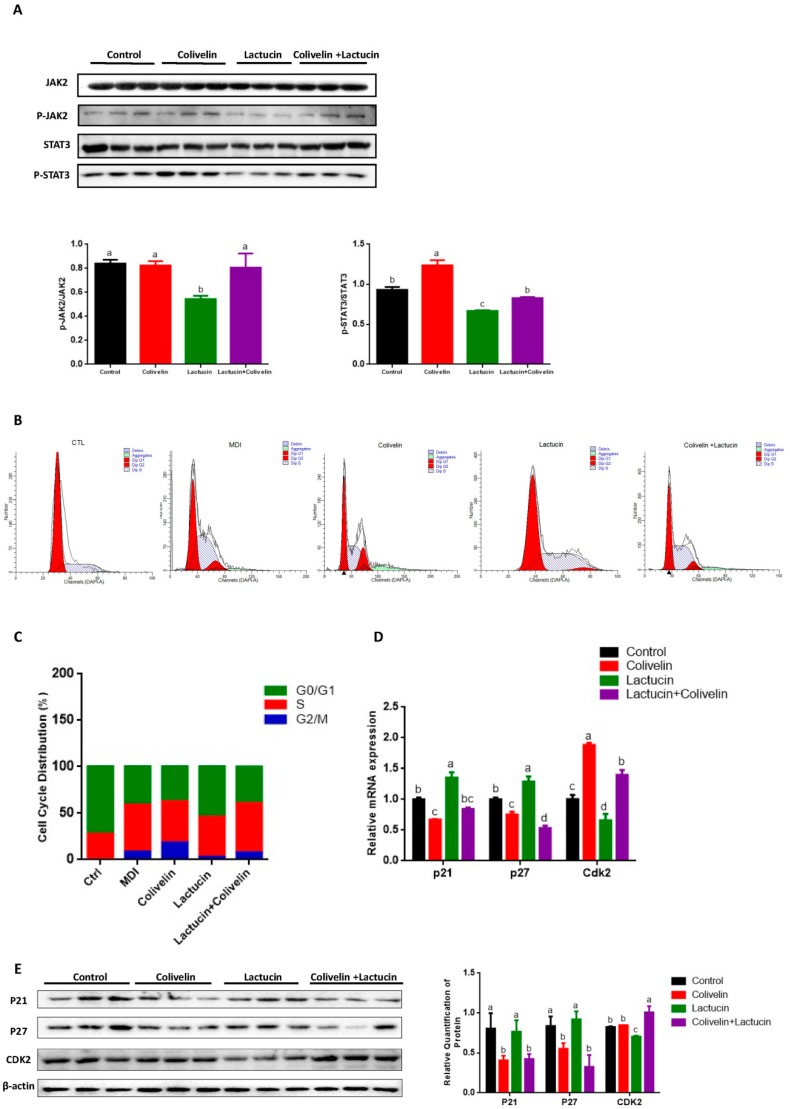
The pharmacological STAT3 activator reverses the cell cycle arrest of lactucin on 3T3-L1 cells. The 3T3-L1 preadipocytes were induced to differentiate in the presence or absence of lactucin and colivelin for 24 h. (**A**) The immunoblot analysis of JAK2/STAT3 signaling pathway in 3T3-L1 adipocytes after treatment (*n* = 3). (**B**) The cell cycle analysis using flow cytometry. (**C**) Percentage of cells in G0/G1, S and G2/M phases measured using FlowJo V10 software. (**D**,**E**) The mRNA and protein levels of genes involved in cell cycle progression (*n* = 3). Data expressed as mean ± SEM. Statistics were analyzed by ANOVA (Tukey multiple comparisons test), similar letters (a, b, c, d) indicate the absence of significant differences.

To further test whether lower levels of phospho-JAK2/STAT3 was the cause of lactucin-mediated anti-adipogenesis, cells were treated with colivelin, a specific activator of STAT3 [34,35,36]. First, the activation of STAT3 by colivelin was examined. The results showed that compared with the lactucin group, colivelin treatment significantly upregulated the phosphorylation of JAK2 and STAT3 (*p* < 0.05) (Figure 5A), suggesting the effectiveness of colivelin in the activation of JAK2/STAT3 signaling pathway. Then, we analyzed the cell cycle changes by flow cytometry, the result showed that compared to lactucin-treated cells, colivelin treatment significantly attenuated the arrestment of G0/G1 phase by lactucin (Figure 5B,C). Consistently, compared to lactucin-treated cells, colivelin treatment significantly decreased expression levels of p21 and p27, and correspondingly increased the mRNA and protein levels of CDK2 (Figure 5D,E).

**Figure 6:**
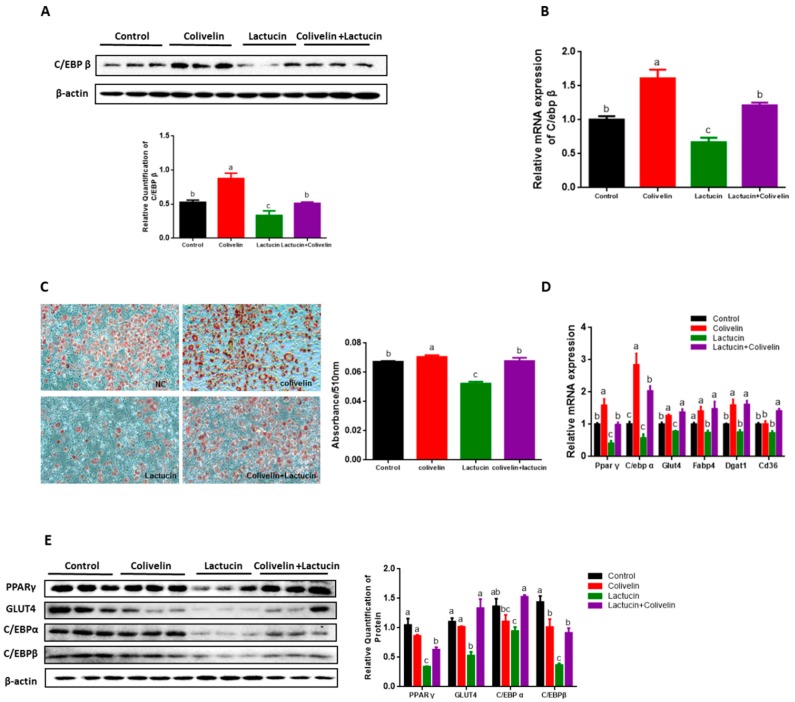
The pharmacological STAT3 activator reverses the anti-adipogenic effect of lactucin on 3T3-L1 cells. (**A**) The immunoblot analysis of C/EBP β in 3T3-L1 adipocytes after treatment (*n* = 3). (**B**) The mRNA level of C/ebp β in 3T3-L1 adipocytes after treatment (*n* = 3). (**C**) Oil Red O staining of 3T3-L1 adipocytes photographed using a microscope (×100). Lipid droplets were stained in red. And absorbance of extracted ORO accumulated in lipid droplets of 3T3-L1 adipocytes was measured spectrophotometrically at 510 nm. (**D**) The mRNA levels of lipid metabolism genes in 3T3-L1 adipocytes after treatment (*n* = 3). (**E**) The immunoblot analysis of lipid metabolism genes in 3T3-L1 adipocytes after treatment (*n* = 3). Data expressed as mean ± SEM. Statistics were analyzed by ANOVA (Tukey multiple comparisons test), similar letters (a, b, c) indicate an absence of significant differences.

Next, we noticed that colivelin treatment significantly attenuated the inhibitory effect of lactucin on adipogenesis, showing less lipid accumulation in lactucin-treated cells than vehicle-treated controls, but a similar amount of lipid in addition with colivelin treatment (*p* < 0.05) (Figure 6C). Consistently, compared to lactucin-treated cells, colivelin treatment significantly increased the mRNA and protein levels of PPAR γ, C/EBP α, C/EBP β, GLUT4 and FABP4 (*p* < 0.05) (Figure 6A,B,D,E).

Together, these results demonstrate that activation of STAT3 by colivelin alleviates the inhibitory effect of lactucin on adipogenesis of 3T3-L1 cells, suggesting lactucin exerts anti-adipogenesis effect through downregulation of JAK2/STAT3 signaling pathway.

## 4. Discussion

Obesity is a major worldwide issue that increases the incidence of type 2 diabetes mellitus, cardiovascular diseases, hepatosteatosis, and other chronic diseases [37,38]. Therefore, developing an effective way to control adiposity is an important goal in metabolic disease research. Considerable efforts have been made to investigate the anti-obesity therapeutic targets. However, most of the anti-obesity agents reported to date have side effects, such as diarrhea, vomiting, nausea, and insomnia [39]. To overcome these side effects, many scientists have sought to identify natural compounds that have antiadipogenic effects [40,41]. Lactucin, a natural sesquiterpene lactone, with anti-malaria and anti-hyperalgesia effect. In the present study, we for the first time revealed a previously undiscovered anti-obesity function of lactucin in mice and in 3T3-L1 cells. Our results open a new avenue of exploring potential safe and effective anti-obesity agents.

The phenotype of lactucin-treated mice and adipocytes is less lipid accumulation. The cause of a decrease in lipid accumulation was at least partially attributed to the reduction of TG synthesis as evidenced by lower levels of TG synthesis markers and glucose uptake into adipocytes. Consistent with these results, several studies have shown that most sesquiterpene lactone compounds such as Zaluzanin C [20], Dehydroleucodine (DhL) [21] and Artesunate [22] also suppress the adipocyte differentiation via inhibiting lipid synthesis. However, we showed a characteristic feature of lactucin different from other anti-lactone compounds: it inhibits adipogenesis in the early stages of adipocyte differentiation. Normally, when preadipocytes are exposed to adipogenic inducers, adipocyte differentiation occurs [42]. Confluent preadipocytes initiate MCE by adipogenic signals and re-enter cell cycle progression followed by growth arrest [43]. The cells then become spherical, accumulate lipid droplets, and develop into fully differentiated cells [44]. Therefore, Lactucin has the potential to be explored as a food additive or natural health product for the prevention of obesity in early development.

Mechanistically, our observations showed that Lactucin inhibited MCE by inducing G0/G1 phase arrest during the early stage of adipocyte differentiation by flow cytometry analysis. We further demonstrated that lactucin blocks MCE at the early stage of adipocyte differentiation, as evidenced by a significant reduction in Cyclin-dependent kinase 2 (CDK2) levels with concomitant and sustained expression of p27 and p21. Previous studies indicated that the constitutive overexpression of the cell-cycle inhibitor p27 prevents cells from entering the S-phase of the cell cycle and thereby disrupts all subsequent steps of differentiation. [45,46]. Of note, we found changes in CCAAT/enhancer-binding protein β (C/EBP β) (Figure 3B), which is required for MCE during adipogenesis via DNA binding activity followed by expression of adipocyte and cell cycle markers [47,48]. Meanwhile, the expression of PPAR γ and C/EBP α, the subsequent targets of C/EBP β, was prevented with lactucin treatment. These investigations indicate that lactucin-induced MCE arrest during the early stage of adipogenesis might result from a decrease of C/EBP β in preadipocytes. In this study, we found that the JAK2/STAT3 signaling pathway was inhibited, which may be the cause for the reduction of C/EBP β. Several studies suggest that the JAK2/STAT3 pathway is involved in the early stage of 3T3-L1 preadipocyte differentiation through regulating the C/EBP β transcription, and C/EBP β is the target genes of STAT3 [32,48]. The further results show colivelin, a STAT3 activator, efficiently rescues Lactucin-induced MCE arrest and promotes lipid accumulation in 3T3-L1 adipocyte, supporting the hypotheses that the Lactucin induced MCE arrest through downregulation of the JAK2/STAT3 pathway. To be noted, several studies have shown that the JAK2-STAT3 signaling pathway is downstream of leptin [49,50]. Changes in this pathway may reflect the potential involvement of leptin. We exclude the involvement of leptin in the action of Lactucin based on similar food intake between two groups of mice since the effect of leptin is to suppress appetite.

In conclusion, the present study indicates that lactucin ameliorates high-fat diet-induced obesity through inhibiting TG synthesis in adipose tissue of mice. Also, lactucin inhibits adipogenesis in vitro through arresting MCE during the early stage of adipocyte differentiation by inhibiting the JAK2/STAT3 pathway. Therefore, lactucin has the potential to be explored as a food additive or nutraceutical candidate for preventing/treating obesity and metabolic diseases. Further studies are necessary to comprehensively evaluate the safety and the pharmacokinetics of lactucin in more advanced animal models and/or humans.

## Supplementary Materials

The following are available online at https://www.mdpi.com/2073-4409/9/2/331/s1, Figure S1: Lactucin does not cause toxicity in mice. Figure S2: The effect of lactucin on hepatic lipogenesis. Table S1: Primers used in real-time quantitative PCR. Table S2: Anti-body used in western-blot and immunofluorescence.
