# Supplementary material for: A Potential Nutraceutical Candidate Lactucin Inhibits Adipogenesis through Downregulation of JAK2/STAT3 Signaling Pathway-Mediated Mitotic Clonal Expansion
Source: Cells. 2020 Jan 31;9(2):331. doi: 10.3390/cells9020331 (PMC7072480; doi:10.3390/cells9020331)
Supplement: Supplementary file 1 [file cells-09-00331-s001.pdf]

Supplemental Figure 1

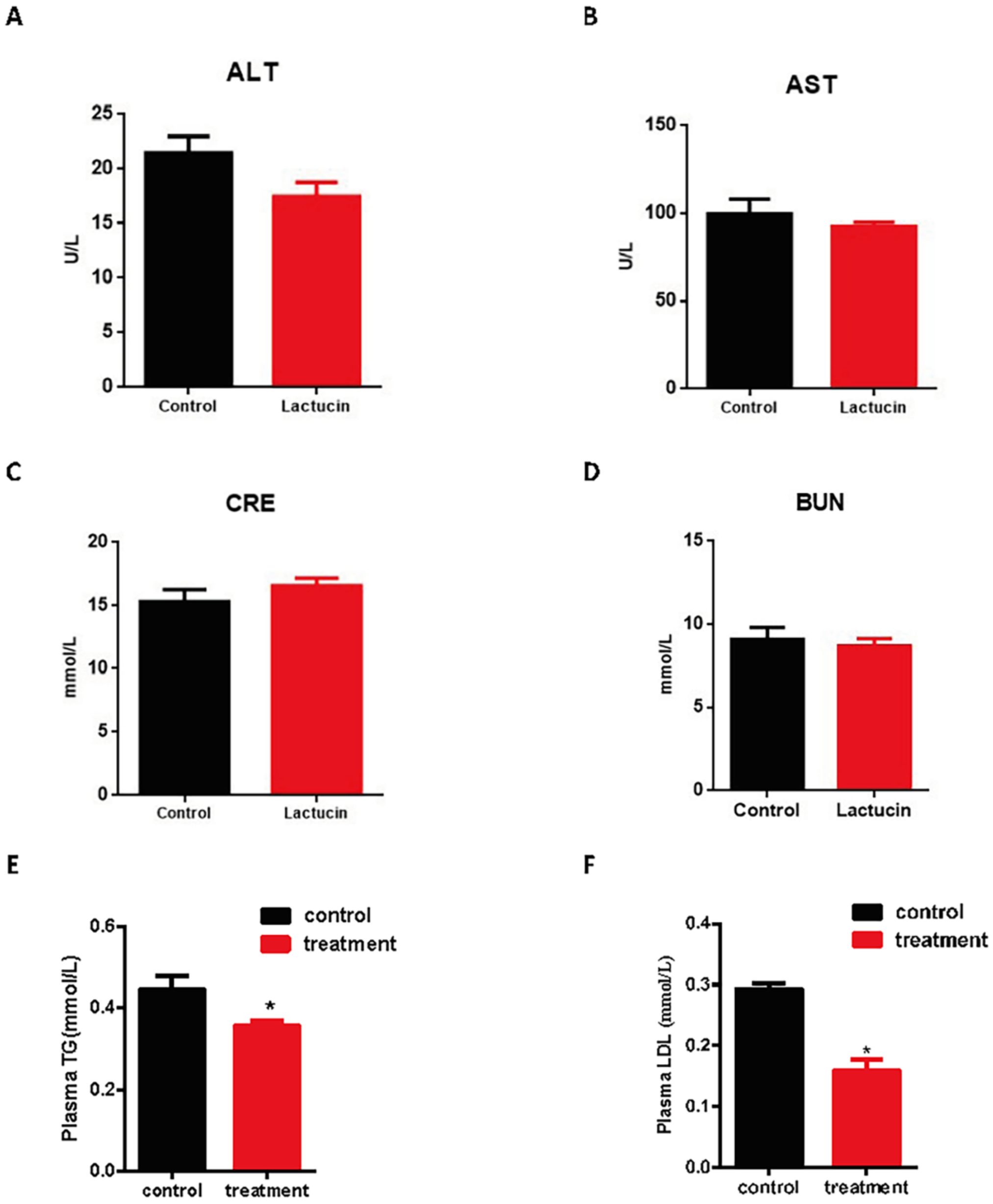

Supplemental Figure 1. (A-B) Levels of plasma alanine aminotransferase (ALT) and aspartate aminotransferase (AST) (n=6). (C-D) Levels of plasma urea nitrogen (BUN) and creatinine (CREA) (n=6). (E-F) Levels of plasma TG and LDL. Values are mean ± S.E.M. * $p < 0.05$, ** $p < 0.01$, ***$p < 0.001$.

Supplemental Figure 2

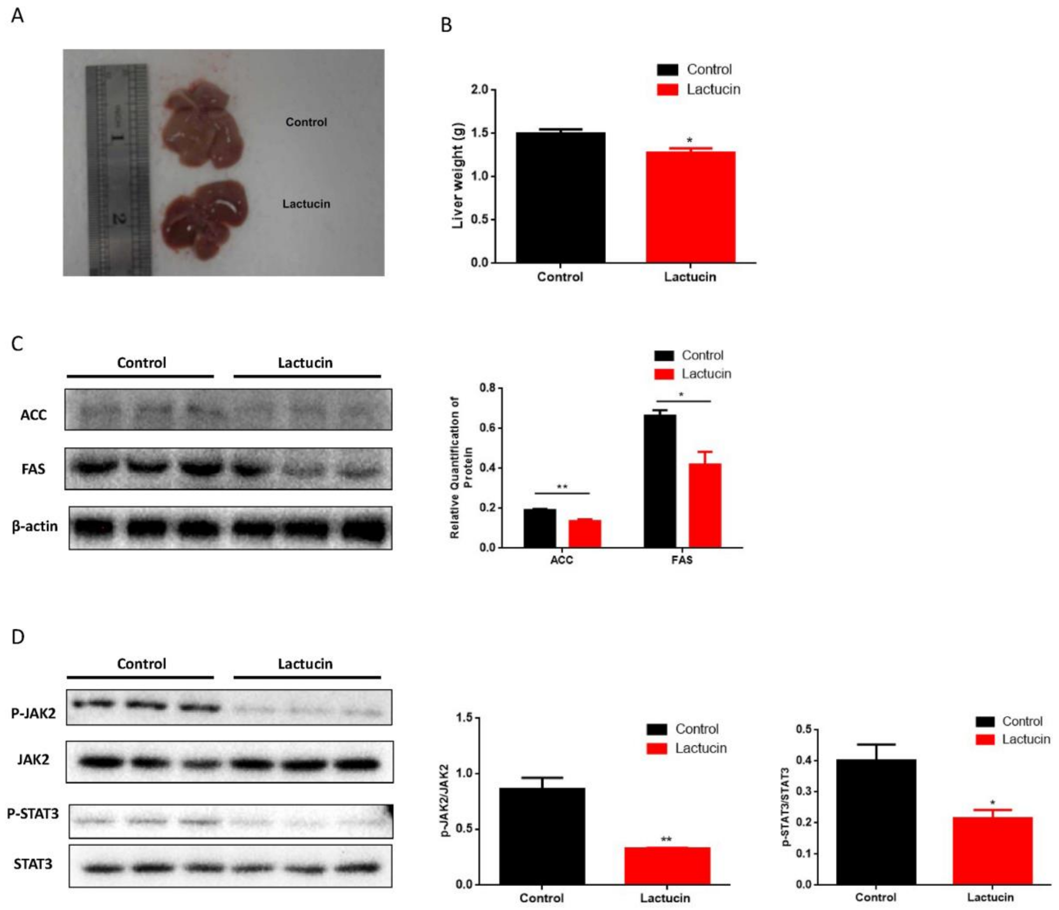

Supplemental Figure 2. (A) Morphology of the liver. (B) The weight of liver (n=7). (C) The immunoblot analysis of ACC and FAS in liver (n=3). (D) The immunoblot analysis of JAK2/STAT3 signaling pathway in adipose tissue of mice treated with vehicle or lactucin (n=3). Values are mean ± S.E.M. * $p < 0.05$, ** $p < 0.01$, ***$p < 0.001$.

Supplemental Tables

Supplemental Table 1. Primers used in real-time quantitative PCR.

Target gene	Forward (5′-3′)	Reverse (5′-3′)
β-Actin	GTCCCTGACCCTCCCAAAAG	GCTGCCTCAACACCTCAACCC
CCAAT Enhancer Binding Protein β (C/EBP β)	AAGCTGAGCGACGAGTACAAGA	GTCAGCTCCAGCACCTTGTG
Peroxisome proliferator activated receptor γ (PPAR γ)	CCAAGAATACCAAAGTGCGATCA	CCCACAGACTCGGCACTCAAT
CCAAT Enhancer Binding Protein α (C/EBP α)	TGGACAAGAACAGCAACGAG	TCACTGGTCAACTCCAGCAC
Diacylglycerol O-Acyltransferase 1 (DGAT 1)	CCGTGTTTGCTCTGGCATC	TGACCTTCTTCCCTGTAGAG
Diacylglycerol O-Acyltransferase 2 (DGAT 2)	CCTTCCTGGTGCTAGGAGTG	CCAGTCAAATGCCAGCCA
adipocyte protein 2 (aP2)	AAGAAGTGGGAGTGGGCTTTG	CTCTTCACCTTCCTGTCGTCTG
Glucose transporter 4 (Glut4)	CTGATTCTGCTGCCCTTCTGTCCT	GACATTGGACGCTCTCTCTCCAACTT
CD36 Molecule (Cd36)	CCTTAAAGGAATCCCCGTGT	TGCATTTGCCAATGTCTAGC
p27	GGGCTCGTCTTTTCGGGGTGTTT	GAGCGGGAGGGCGGAGAGGAG
p21	AGTGTGCCGTTGTCTCTTCG	ACACCAGAGTGCAAGACAGC
Cyclin-dependent kinase 2 (CDK 2)	AGGCATGAGGAATCTGGGAG	GAGGTGGACGTCAGAGGAAA

Supplemental Table 2. Anti-body used in western-blot and immunofluorescence.

Antibody	From	Dilution (WB/IF)
β-Actin	Cell signaling Technology (4970S)	1:5000
CCAAT Enhancer Binding Protein β (C/EBP β)	Santa Cruz Biotechnology (sc-7962)	1:1000/1:250
CCAAT Enhancer Binding Protein α (C/EBP α)	Santa Cruz Biotechnology (sc-365318)	1:1000
CD36 Molecule (Cd36)	Cell signaling Technology (14347S)	1:1000
Peroxisome proliferator-activated receptor gamma coactivator 1-alpha (PGC1 α)	Cell signaling Technology (2179S)	1:1000
Peroxisome proliferator activated receptor γ (PPAR γ)	Cell signaling Technology (2430S)	1:2000
Fatty acid binding protein 4 (FABP4)	Cell signaling Technology (2120S)	1:1000
Acetyl-CoA carboxylase (ACC)	Cell signaling Technology (3662)	1:1000
Fatty acid synthase (FAS)	Cell signaling Technology (3180)	1:1000
Cyclin-dependent kinase 2 (CDK 2)	Santa Cruz Biotechnology (sc-6268)	1:1000
p27	Santa Cruz Biotechnology (sc-1641)	1:1000
p21	Santa Cruz Biotechnology (sc-6246)	1:1000
Glucose transporter 4 (Glut4)	Abcam (ab654)	1:2000/1:250
Janus kinase 2 (JAK 2)	Wanleibio (WL02188)	1:500

Phospho JAK 2 (Tyr1007/1008)	Wanleibio (WL02997)	1:500
Signal transducer and activator of transcription 3 (STAT3)	Wanleibio (WL01836)	1:500
Phospho-Stat3 (Tyr705)	Wanleibio (WLP2412)	1:500
Goat anti-rabbit (FITC)	Abbkine A23420	1:1000
Goat anti-mouse (FITC)	Abbkine A23210	1:1000
